# Changes in Poly(ADP-Ribosyl)ation Patterns in Workers Exposed to BTX

**DOI:** 10.1371/journal.pone.0106146

**Published:** 2014-09-12

**Authors:** Yan Sha, Wei Zhou, Zhenyu Yang, Xiaoling Zhu, Yingping Xiang, Tiandi Li, Dexiang Zhu, Xinyue Yang

**Affiliations:** 1 Department of Education and Research, Shenzhen Prevention and Treatment Center for Occupational Diseases, Shenzhen, Guangdong, China; 2 Department of Occupational Hazard Assessment, Shenzhen Prevention and Treatment Center for Occupational Diseases, Shenzhen, Guangdong, China; 3 Physicochemical Laboratory, Shenzhen Prevention and Treatment Center for Occupational Diseases, Shenzhen, Guangdong, China; 4 Department of Integrated Services, Shenzhen Prevention and Treatment Center for Occupational Diseases, Shenzhen, Guangdong, China; Tel-Aviv University, Israel

## Abstract

Occupational exposure to (benzene, toluene and xylene, BTX is common in the Chinese workplace. Chronic occupational exposure to benzene is associated with an increased risk of hematological malignancies such as acute myeloid leukemia (AML), but the underlying mechanisms are still unclear. This study investigates changes in poly(ADP-ribosyl)ation and DNA methylation in subjects occupationally exposed to a BTX. Blood DNA samples and exposure data were obtained from subjects with different levels of exposure, including 132 decorators, 129 painters, and 130 unexposed referents in a container-manufacturing factory in Shenzhen, China. Occupational exposure assessment included personal monitoring of airborne benzene, toluene and xylene. Hematological parameters were measured and the cytokinesis-block micronucleus (CBMN) assay was used to detect DNA damage in peripheral lymphocytes. Quantitative real-time PCR was used to detect the mRNA expression of poly(ADP-ribose) polymerase 1 (PARP1) and poly(ADP-ribose) glycohydrolase (PARG), DNA methyltransferases (DNMTs) including DNMT1, DNMT3a and DNMT3b, methyl-CpG-binding domain protein 2(MBD2). PARP1 assay was used to measure PARP activity. Airborne levels of benzene, toluene and xylene in the two exposed groups were significantly higher than those of controls (*P*<0.001). The two exposed groups (decorators, painters) showed decreased PARP1, DNMTs and MBD2 expression relative to controls (*P*<0.05), and PARP activity was also decreased (*P*<0.05). Decreased PARP1, DNMT1, DNMT3a, DNMT3b and MBD2 mRNA expression was correlated with increased airborne BTX (Pearson's r: −0.587, −0.314, −0.636, −0.567 and −0.592 respectively, P<0.001). No significant differences in hematological parameters and CBMN were found among the three groups. Together, these results suggest that decreased DNMTs, MBD2 and PARP1 might be involved in the global hypomethylation associated with BTX exposure, and the imbalance of PARP/PARG might participate in the down-regulation of DNMTs. This is the first human study to link altered poly(ADP-ribosyl)ation patterns, which reproduce the aberrant epigenetic patterns found in benzene-treated cells, to chronic occupational exposure to BTX.

## Introduction

Mixtures of benzene, toluene and xylene (BTX) are very commonly encountered solvents in the Chinese workplace. The concentrations of benzene or BTX have a broad range (124.8 mg/m^3^ for leather products, 98.7 mg/m^3^ for electronic devices, 75.4 mg/m^3^ for machinery, 50.4 mg/m^3^ for shoes, and 50.3 mg/m^3^for office supplies and sports equipment manufacturing) [2], [3], [4], [5].

Benzene, toluene and xylene are monocyclic aromatic hydrocarbon compounds. Studies involving exposures to these compounds individually have shown that benzene poses maximum hazard to the exposed organism through ROS generation in comparison to toluene and xylene. [6]. Benzene is classified as a human carcinogen by the International Agency for Research on Cancer [7]. It is assumed that toluene and xylene may interact and determine the toxicity of the benzene fraction by influencing toxicokinetics [1].

Occupational exposure to benzene is of great concern because of its carcinogenicity, even at low concentrations. Exposure levels in the Chinese workplace typically are below the Occupational Exposure Limit of 6 mg/m^3^ (8 h time-weighted average) [8], but chronic benzene poisoning still exists. For example, in the past 4 years, 26 cases of occupational chronic benzene poisoning cases have been diagnosed in Shenzhen City in recent 4 years. Whether there is a safe level of benzene exposure has been highly debated.

Benzene was shown to induce hematotoxicity in humans over 100 years ago and recent studies have reported hematological effects at various benzene levels including as low as 1 p.p.m. in air [9], [10]. Benzene is a cause of acute myeloid leukemia (AML) and myelodyplastic syndrome, and a probable cause of other hematological malignancies, such as non-Hodgkin lymphoma [11], [12]. In AML and other cancer tissues, aberrant DNA methylation patterns, including global hypomethylation, gene-specific hypermethylation or hypomethylation, and loss of imprinting (LOI), are common [13], [14]. As in some other malignancies, global genomic DNA methylation levels tend to decrease in each step of the progression from normal to AML cells [15].

Genomic-wide DNA hypomethylation has been reported in subjects exposed to low-level benzene [16], [17] but the molecular pathogenesis is unknown. One possible mechanism is intereference with poly(ADP-ribosyl)ation (PARylation), which participates in the establishment and maintenance of the genome's methylation pattern [18], [19]. PARylation is a post-translational modification involving the polymerization of ADP-ribose (ADPR) units from donor NAD^+^ molecules on target proteins [20]. The synthesis and degradation of poly(ADP-ribose) (PAR) is catalyzed by two types of enzymes: PAR polymerases (PARPs) and PAR glycohydrolases (PARGs), respectively [21]. PARP-1, a ubiquitous 116-kDa nuclear enzyme, is the founding member of the PARP superfamily [21]. It is a highly abundant protein (1–2 million molecules per cell) that is likely responsible for the majority of PAR synthesis in cells [20].

We hypothesized that DNA methylation transferences (DNMTs) and poly(ADP-ribosyl)ation are changed in subjects with chronic BTX exposure. This would presumably be a persistent effect because, to date, no DNA demethylase enzyme has been found. The study was therefore designed to evaluate 2 BTX-exposed populations with a single group of individuals with no occupational exposure to BTX. We assessed and compared (a) hematological parameters, (b) mRNA expression of PARP1, PARG and PARP activity, and (c) mRNA expression of DNA methylation transferases (DNMT).

## Materials and Methods

### Ethics statement

The study was approved by Institutional Review Boards at the Shenzhen Prevention and Treatment Center for Occupational Disease (Permit Number 2012021). Participation was voluntary, and written informed consent was obtained.

### Study population

The study population consisted of 132 decorating and 129 painting workers employed at a container-manufacturing plant in south China and 130 unexposed office workers, used as controls, from the same plant. Controls were frequency matched by sex and age to exposed workers. Self-administered questionnaires collected information regarding exposure history, medical history, lifestyle factors, smoking habits and other personal information. Peripheral bloods were collected.

### Environmental monitoring

Firstly we make qualitative determination of the paint in the workplace by gas chromatography-mass spectra detector. According to the GS-MS result, environmental monitoring for benzene, toluene and xylene in the breathing zone of workroom air was performed according to Determination of Toxic Substances in Work place air-benzene and its chemical compounds (GBZ/T 160.43-2004, China). A Galian LFS-113 individual air sampler (Galian, USA, flow rate 50 cm^3^/min) placed near the subject's breathing zone was used to collect air samples with a carbon tube during a typical work-shift (midweek, 8:00 a.m. to 12:00 p.m.). The chemicals were determined by CS_2_ desorption followed by Agilent 7089A gas chromatography, using standard curve method; the limit of detection for benzene, toluene, and xylene were 0.01 mg/m^3^, 0.3 mg/m^3^, and 0.3 mg/m^3^, respectively.

All subjects received a health examination, including an interview by a doctor or a public health nurse for medical history and completion of questionnaires, and a clinical examination according to the Chinese technical specifications for occupational health surveillance for benzene and its chemical compounds (GBZ188-2007).

### Blood sampling

Blood samples (10 mL) obtained by venipuncture were divided in two aliquots: one portion was put into ethylenediamine–tetraacetic acid tubes for assessment of hematological markers and mRNA expression (5 mL); the other was treated with heparin and used for the MN assay and PARP1 activity assay (5 mL). Processing and scoring of the three sample groups were performed, immediately blind, and concurrently. At the end of the study, questionnaire data, exposure records and blood parameters were linked to unique code numbers for data analysis.

### Analysis of hematological markers

Blood samples were delivered to the laboratory within 3 hours of collection. Complete blood count and differential assays were determined with a Beckman-Coulter T540 blood counter.

### Real time quantitative PCR

mRNA expression of PARP-1, PARG, DNMT1 were quantified using real-time quantitative PCR. In brief, total RNA was extracted from blood using a total RNA rapid exaction kit (Bioteke, Beijing, China) according to the manufacturer's protocol. RNA quantity and quality were evaluated by UV spectrophotometry and denaturing agarose gel electrophoresis, and 2 µg total RNA was reverse-transcribed by the PrimeScript RT reagents kit (Takara, Dalian, China) according to the manufacturer's instructions. cDNAs were analyzed immediately or stored at −20°C until use. Real-time PCR assay was carried out using the Applied Biosystems 7900 HT Fast Real-Time PCR System (Applied Biosystems, Inc., Foster City, CA, USA), using SYBR Premix Taq (Takara, Dalian,China), to observe the mRNA levels of targeted genes.β-actin was used as an endogenous control to normalize expression levels. The length and sequence of PCR products were identified by melting curve analysis, and the band size was confirmed using 2% agarose electrophoresis. Primers of the target fragments were designed and synthesized according to Table 1.

**Table 1:** Primers used in quantitative real time PCR.

Sequence ID	Forward primer (5′ to 3′)	Reverse primer (5′ to 3′)
*PARP-1*	CCCAGGGTCTTCGGATAG	AGCGTGCTTCAGTTCATACA
*PARG*	CAGTCCATCTTGCCTGAT ATGGTG	CAATCTGTTCCTGCgGACATTGTG
*DNMT1*	ACGACCCTGACCTCAAATAT	CCATTAACACCACCTTCAAGA
*DNMT3a*	CACAGAAGCATATCCAGGAG	CACATTCTCAAAGAGCCAGA
*DNMT3b*	AGTATCAGGATGGGAAGGAG	CGATAGGAGACGAGCTTATTG
*MDB2*	ACTATAAGTGCCCTCTGTGT	TCAGAGTCTCCTTCATGTACTT
β-actin	TGGCACCCAGCACAATGAA	CTAAGTCATAGTCCGCCTAGAAGCA

The experimental reaction was achieved by adding the following components sequentially: Nuclease-free PCR-grade water 6.8 ul; cDNA 2.0 ul; SYBR Premix Taq 10.0 ul; forward primer (10 uM) 0.4 ul; reverse primer (10 uM) 0.4 ul; ROX Reference Dye(50×) 0.4 ul; total reaction volumes were 20 ul; and performed PCR in 7900 HT under the following conditions: 95°C for 30 s for 1 cycle, followed by 40 cycles of 95°C for 5 s, and 60°C for 30 s. All experiments were independently performed in triplicate; no template control wells were used as negative control. RQ Manager 1.2 software was used to obtain gene-related expression levels.

### CBMN assay

The lymphocyte CBMN assay using whole blood cultures was performed as described by Fenech [22], [23]. Five mL venous blood was collected in lithium heparin tubes. Whole blood cultures of lymphocytes were prepared in RPMI 1640 (Sigma) containing 10% fetal bovine serum (GIBICO, USA) 2 mM l-glutamine (Sigma), 1 mM sodium pyruvate (Trace Scientific) as previously described and kept at 37°C and 5% CO_2_ in a humidified incubator. Duplicate cultures were set-up for each subject. Cytochalasin-B was added after 44 h of culture, cells were harvested 28 h later and then fixed and stained. Frequency of BN cells with one or more micronuclei (MN-BN) was determined by scoring 1000 BN cells from each duplicate culture using established scoring criteria. Coefficient of variation of duplicate measurements of MN-BN was 11.1%.

### PARP activity assay

Peripheral lymphocytes were isolated from heparinized blood by Histopaque gradient centrifugation (Sigma, St. Louis, MO, USA). Cells lysed in ice-cold PARP buffer (including 0.4 mmol/L PMSF, 0.4 mol/L NaCl, 1% triton X-100) were collected. After incubation for 30 min on a rotator at 4°C, cell lysates were centrifuged to remove cell debris. Protein concentrations were determined with detergent-compatible reagent (Bio-Rad). PARP activity was determined using the Universal Colorimetric PARP Assay Kit (Trevigen lnc, USA) according to the manufacturer's instructions.

### Statistical analysis

One-way ANOVA was employed to test the differences among the two exposed groups and control group for the gene expression level of PARP1, PARG, DNMT1, DNMT3a, DNMT3b, MBD2 and PARP activity. Relationships between the gene expression and airborne benzene level were calculated using Pearson correlations. All statistical analyses were performed with SPSS 16.0 for windows (SPSS Inc., Chicago, IL). A *P*-value <0.05 was considered statistically significant.

## Results

There were no significant differences in sex, smoking and alcohol consumption among the exposed workers and control group. The study participants were all male, and most of the individual characteristics were similar in both exposed workers and controls (Table 2). All exposure indices were significantly higher in workers than controls, and the exposure level of painters was higher than decorator (*P*<0.001). Controls were exposed to benzene mixture levels below the limit of detection (LOD).

**Table 2:** Characteristics and airborne chemical concentration in the control and BTX-exposed groups.

	control	BTX-exposed subjects		*P*
	Office workers (n = 130)	Decorators (n = 132)	Painters (n = 129)	
Mean age, y(min-max)	31.04±6.25	30.82±6.18	32.06±6.16	0.22
Gender				
Male	120	120	121	
Female	10	12	9	0.68
Job-years	5.3±3.1	5.6±4.9	6.3±5.1	0.23
Personal protective equipment	no	gas mask	gas mask	
Cigarette smoking, n (%)				
Never	56 (43%)	64 (48%)	58 (45%)	
Former smoker	27 (21%)	35 (27%)	39 (30%)	
Current smoker	47 (36%)	33 (25%)	32 (25%)	0.16
Cigarettes/day, n (SD)	8.9 (4.5)	7.5(3.8)	8.6(4.9)	0.35
Median personal benzene exposure, mg/m^3^ (IRQ)	0.005	0.03(0.02–0.04)	0.21(0.12–0.32)	<0.001*
Median personal toluene exposure, mg/m^3^ (IRQ)	0.15	5.3(4.1–6.0)	21.5(17.9–26.7)	<0.001*
Median personal xylene exposure, mg/m^3^ (IRQ)	0.15	3.6(3.1–4.3)	26.4(19.0–28.4)	<0.001*

Abbreviations: IQR, interquartile range; SD, standard deviation.

*P<0.05.

*P* values were obtained from Pearson's chi-square test for categorical variables and ANOVA for continuous variables.

Because all controls have values less than LOD for benzene, toluene, and xylene of 0.01, 0.3, 0.3 mg/m^3^ respectively, these data were assigned a value corresponding to one half of the LOD in the analysis (i.e. 0.005 mg/m^3^ for benzene).

Categorical variables are expressed as n (%), and continuous variables are expressed as mean ± SD.

There were no significant differences in blood counts between the exposed groups and control group (Table 3). Clinical examination was within normal limits in both the two exposed and control groups.

**Table 3:** Comparison of blood test results among the BTX-exposed groups and the control group.

Items	Control	Decorators	Painters	*F*	*P*
n	130	132	129		
WBC (×10^9^/L)	6.76±1.55	6.68±1.57	6.47±1.40	0.451	0.638
RBC (×10^12^/L)	5.16±0.68	5.04±0.31	5.09±0.28	0.978	0.378
Hb (g/L)	149.7±9.46	151.2±11.83	153.1±8.78	1.263	0.286
Platelet (×10^9^/L)	231.2±38.8	238.9±52.3	239.9±45.5	0.458	0.633

*P* value was obtained from one-way ANOVA.

### Micronuclei in Cytokinesis-Blocked Cells

The incidence of MN in cytokinesis-blocked T-lymphocytes of all study subjects was determined following the standard CBMN protocol. No significant difference was observed comparing the two exposed groups and the control (Table 4).

**Table 4:** MN in peripheral lymphocytes in subjects grouped by exposure and smoking habits.

Group	Subjects (n)	MN/1000BN cells	
		Mean ± S.D.	95% CI
Controls			
Total	130	1.51±0.59	1.41–1.62
Never	56 (43%)	1.44±0.56	1.29–1.59
Former smoker	27 (21%)	1.50±0.50	1.30–1.70
Current smoker	47 (36%)	1.61±0.67	1.42–1.81
Decorators			
Total	132	1.56±0.65	1.45–1.67
Never	64 (48%)	1.58±0.73	1.40–1.76
Former smoker	35 (27%)	1.53±0.48	1.37–1.70
Current smoker	33 (25%)	1.55±0.65	1.32–1.78
Painters			
Total	129	1.53±1.51	1.44–1.62
Never	58 (45%)	1.48±0.48	1.35–1.60
Former smoker	59 (30%)	1.55±0.46	1.40–1.70
Current smoker	32 (25%)	1.40±0.16	1.35–1.46

Abbreviations: BN, binucleated cells.

No significantly different between exposed and control.

### Poly (ADP-ribosyl)ation patterns

**Figure 1:**
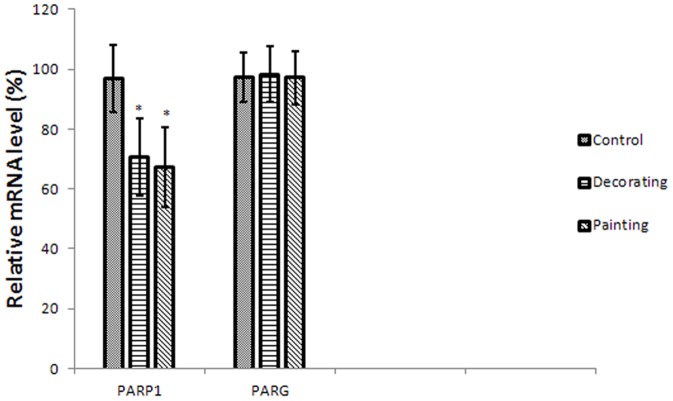
PARP-1 expression in exposed groups and control. mRNA level was measured by real-time PCR with input normalization by ACTB mRNA level. The results are expressed as percentage of the controls (mean ± SD) from three independent experiments. *P<0.05 versus control.

Gene-specific analysis showed PARP1 mRNA expression decreased significantly in the decorator group and painter groups (*P*<0.05). PARG mRNA expression showed a moderate non-significant decrease in painters, with no change in decorators, compared with referents (Figure 1).

**Figure 2:**
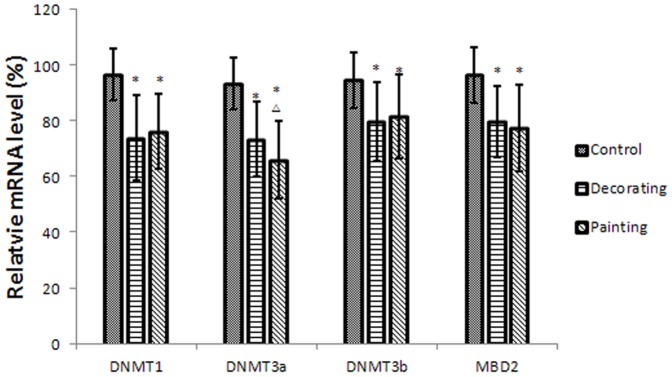
DNMTs and MBD2 expression in exposed groups and control. mRNA level was measured by real-time PCR with input normalization by ACTB mRNA level. The results are expressed as percentage of the controls (mean ± SEM) from three independent experiments. *P<0.05 versus control and a dose-dependent decrease in gene expression was observed in DNMT3a (*P*<0.05).

The remarkable decrease in PARP1 mRNA in BTX-exposed workers led us to examine the effect of exposure on PARP activity in subjects' peripheral blood. We found a significant decrease of PARP1 activity in the two exposed groups (*P*<0.05, Figure 2), indicating PARP-1 in responsible for the PARP activity.

### Expression of DNMTs and MBD2

**Figure 3:**
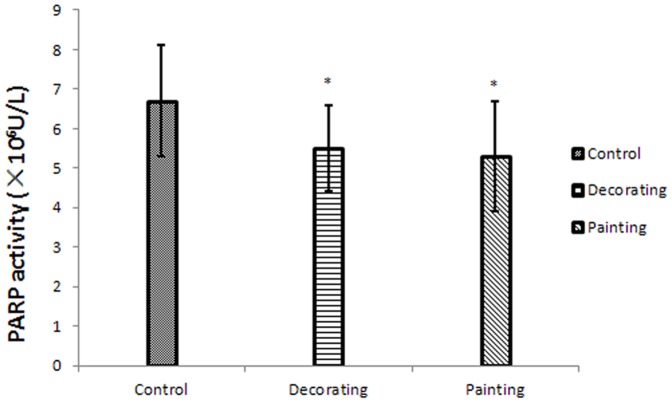
PARP activity in exposed groups and control. PARP activity was determined using the Universal Colorimetric PARP Assay Kit. *P<0.05 versus control.

We determined expression levels of DNMTs and MBD2 as a measure of genomic DNA methylation status. qRT-PCR analysis showed that all of DNA methytransferases were down-regulated in the two exposed groups. Dose-dependent decreases of gene expression were evident in DNMT3a, DNMT3b, and MBD2 (Figure 3).

### Correlation between the level of mRNA expression and BTX exposure

Decreased PARP1 mRNA expression was correlated with increased airborne BTX (Pearson's r = −0.587, P<0.001). In addition, mRNA expression of DNMT1, DNMT3a, DNMT3b and MBD2 were also negatively correlated with airborne BTX (Table 5).

**Table 5:** Correlation between the level of target genes' mRNA expression and BTX exposure.

Gene	Pearson's r	*P*-value
PARG	−0.087	0.087
PARP1	−0.587	0.000*
DNMT1	−0.314	0.000*
DNMT3a	−0.636	0.000*
DNMT3b	−0.567	0.000*
MBD2	−0.592	0.000*

*P<0.05, *P* values were obtained from Pearson correlation.

## Discussions

PARP-1 is an abundant and constitutively expressed nuclear protein that is activated upon the induction of DNA damage by direct binding to DNA breaks through its zinc finger domains [24]. PARP inactivation or cleavage increase genomic instability and accelerates apoptosis. Low expression of the PARP-1 is thought to be involved in carcinogenesis [25]. We used real time RT-PCR and PARP activity to evaluate global poly(ADP-ribosyl)ation patterns. Our results showed BTX exposure-related decrease in the PARP1 mRNA expression and PARP activity. Since poly(ADP-ribose) polymerase 1 (PARP1) is the most abundant isoform of the PARP enzyme family, accounting for about 90% of total cellular PARP activity [26], [27]. Decreased PARP activity may represent an early deviation induced by chronic aromatic hydrocarbon exposure from normal poly(ADP-ribosyl)ation.

We show the mRNA expression of PARP-1 is inhibited in subjects with chronic BTX exposure. However, the mechanisms responsible for low PARP-1 expression have yet to be elucidated. It has been suggested that the epigenetic events can modulate gene expression silence [28]. In vitro study has shown that benzene can induce low PARP-1expression due to the methylation of its promoter in 16HBE cells [29]. Methylation of the PARP-1 promoter is also involved in the regulation of nano-SiO2-induced decrease of PARP-1 mRNA expression in HaCaT cell [30]. These observations suggest that these chemical exposure may induce PARP1 down-regulation through promoter hypermethylation. To the best of our knowledge, this is the first study in humans to link altered poly(ADP-ribosyl)ation patterns and DNA methylation related enzymes to occupational exposure to BTX.

We found that PARP1 mRNA expression decreased significantly, which led to an inbalance of poly(ADP-ribosyl)ation. Zampieri and colleagues have shown that PARG over-expression leads to genomic hypomethylation, which suggests the right balance between PARP/PARG activities maintains the DNA methylation patterns in normal cells [31].

To explore the reason for genomic hypomethylation in workers occupationally exposed to benzene mixtures, we focused attention on the mRNA expression of DNMTs and MBD2.

Five members in the DNMT family have been identified: DNMT1, DNMT2, DNMT3a, DNMT3b and DNMT3L [32]. However, only DNMT1, DNMT3a and DNMT3b interplay to regulate the global DNA methylation pattern. DNMT3b knock-out led to demethylation of minor satellite repeats in embryonic stem (ES) cells, while DNMT1 plus DNMT3b knockout cells displayed a much greater decrease of DNA methylation [33]. Our study showed that all the three DNMTs and MBD2 were down-regulated, indicating that DNMTs and/or MBD2 might participate in the process of DNA methylation in BTX-exposed workers.

We found no significant differences between exposed and control groups in hematological parameters or MN analysis. Others have reported that MN frequency was significantly higher among traffic police than indoor workers, where benzene exposure levels were 0.024 mg/m^3^ and0.004 mg/m^3^, respectively [34]. The possible reasons for the difference with our results might be: firstly, the exposed workers have to wear personal protective equipment. Second, in the workplace, the concentration of toluene, which can decrease benzene metabolism, was much higher than that of benzene. A recent study has shown that adding very high concentrations of benzene alone to HL60 cells can increase reactive oxygen species (ROS) levels and cause cytotoxicity [35], but metabolism of benzene to its toxic metabolites is generally thought to be a critical factor in benzene toxicity [36]. Benzene metabolism and toxicity were reduced in mice following co-administration of high concentrations of toluene, a competitive inhibitor of benzene metabolism [37], although a recent study suggested that toluene enhances the genotoxic effects of benzene in mice at lower doses [38], [39].

In conclusion, our study showed for the first time that low-level occupational exposure to BTX induced in clinically unremarkable workers down-regulation of DNMTs and poly(ADP-ribosyl)ation changes that reproduce the aberrant epigenetic patterns found in benzene-treated cells. Additional studies are required to better define the mechanisms, including the possibility of PARP1 promoter methylation, by which benzene and benzene mixtures produce such alterations. Finally, it should be noted that the aromatic solvent mixtures used in Chinese industry are also commonly employed in the home as solvent for paints. Given the very large number of homes that have been and will continue to be constructed in China, the biological impact of low-level continuous indoor exposure to these agents urgently requires investigation.
